# Extended Dwell Time Improves Results of Fibrinolytic Therapy for Complex Pleural Effusions

**DOI:** 10.7759/cureus.9664

**Published:** 2020-08-11

**Authors:** Sanja H Patino, Francisco Tarrazzi, Catherine Tami, Alyssa Bellini, Mark Block

**Affiliations:** 1 Department of Internal Medicine, Florida Atlantic University Charles E. Schmidt College of Medicine, Boca Raton, USA; 2 Division of Thoracic Surgery, Memorial Healthcare, Hollywood, USA; 3 Department of General Surgery, University of California Davis School of Medicine, Sacramento, USA

**Keywords:** pleural effusion, tissue plasminogen activator, intrapleural therapy, tpa, complex pleural effusion, chest tube

## Abstract

Introduction

Published trials of intrapleural therapy for complex pleural effusions rely on fibrinolytics and deoxyribonuclease (DNase) with dwell times of less than six hours and frequent dosing. We reviewed our experience with fibrinolytics alone but with a longer dwell time (12 hours).

Methods

Tissue plasminogen activator (tPA, 1-6 mg per dose) was given through pigtail catheters placed using image guidance. Planned treatment was for a dwell time of 12 hours with repeat dosing daily for three days or until drainage was less than 100 cc or grossly bloody. Chest x-ray and/or computed tomography (CT) were used to determine completeness of pleural drainage.

Results

Forty-six patients presenting with 47 complex pleural effusions were given 131 doses of tPA. Doses of 4, 5, and 6 mg were most common (n=17, 70, and 33, respectively). Dwell time ranged from five to 14 hours with 12 hours being most common (n=115). Additional chest tubes were placed in 18 effusions. Ten effusions (21%) required decortication: seven for trapped lung and three for incomplete drainage. Drainage was considered complete in 33/40 (82.5%) effusions without trapped lung. Median chest tube duration was seven days (range three to 28 days). tPA therapy was discontinued in two patients for bleeding, but neither experienced hemodynamic instability.

Conclusions

tPA with a 12-hour dwell time is effective and safe for management of complex pleural effusions, although chest tube duration was prolonged. tPA alone is less expensive and easier than when combined with DNase, and this strategy warrants a prospective evaluation.

## Introduction

Intrapleural fibrinolytic therapy can be effective for management of complex pleural effusions that do not respond to thoracentesis [1]. These effusions are typically exudative, with patients presenting in the second, or fibrinopurulent, phase of development [2]. This phase begins about two weeks after the initiating event and lasts for about four to six weeks. Fibrin deposition creates loculated, complex fluid collections that are no longer amenable to simple drainage, ultimately necessitating decortication [1,2]. However, there has been longstanding interest in fibrinolytic therapy instead of surgery when the lung is not "trapped" from organizing fibrosis. Initial experience with intrapleural urokinase and streptokinase supported this concept [3-8]. Tissue plasminogen activator (tPA) is a naturally occurring fibrinolytic, now available as a recombinant protein, and is approved for management of acute thrombotic events such as stroke and coronary artery thrombosis. It is also effective as an intrapleural fibrinolytic agent and recent protocols suggest frequent dosing in combination with DNase for best results [9-11].

Our service frequently manages patients with complex pleural effusions and started using tPA in 2012. DNase was expensive and not readily available, and therefore was not used. Through a serendipitous observation after a chest tube was left clamped overnight, it was discovered that the prolonged dwell time yielded significant drainage. As a result, we implemented a strategy of daily tPA alone with a dwell time of 12 hours. The anecdotal experience seemed encouraging, with the majority of patients achieving effective drainage. Therefore, we undertook a retrospective review to determine if tPA alone with a prolonged dwell time is sufficiently effective to warrant prospective evaluation.

## Materials and methods

Institutional Review Board approval was obtained for this retrospective review. Charts were reviewed for patients with complex pleural effusions that were treated with tPA between November 2012 and April 2016. Demographics and clinical data were collected, and imaging results recorded.

Chest tube placement and tPA therapy

Pigtail catheters (8-14 Fr) were placed into the target effusions using image guidance (CT or ultrasound) and allowed to drain spontaneously. tPA (1-6 mg) in 50 ml of normal saline was given at bedside through the chest tube and the tube was clamped with nursing orders to unclamp after a dwell time of 12 hours. The following day drainage was assessed and a determination was made about whether to repeat treatment. In general, additional doses were given if the drainage was more than 100 ml and not grossly bloody. Target therapy was for three doses over three days. Chest x-ray and chest CT were used to evaluate completeness of drainage. If a residual loculated effusion separate from the drainage catheter was identified, an additional drain was placed and tPA therapy continued. These decisions were made by the individual clinicians and were not protocol-driven.

Outcome assessment

Outcomes were assigned to one of four groups based on imaging: 1) complete drainage, defined as trace to no residual pleural fluid, 2) trapped lung, defined as minimal residual pleural fluid but incomplete expansion of the lung with a pneumothorax replacing the pleural effusion, 3) incomplete drainage without surgery, defined as moderate residual pleural effusion but a clinical decision not to proceed with decortication, and 4) incomplete drainage with surgery, defined as a moderate residual pleural effusion with the clinical decision to proceed with decortication.

## Results

Patient characteristics

Review of medical records identified 46 patients with 47 effusions (one patient had bilateral effusions) treated with at least one dose of tPA (Table 1). Most patients were male, and most effusions were right-sided. Twenty-one effusions were parapneumonic (sterile) and 16 were empyemas with positive pleural fluid cultures (appendix). The most common presenting symptom was dyspnea and many patients had a combination of symptoms (appendix). Symptoms were present on average seven days before initiation of fibrinolytic therapy (range 1 to 30).

**Table 1:** Patient Demographics

Age (years)		
	Mean ± SD	60 ± 13.9
	Range	24 – 92
Gender, n		
	Female	13 (28%)
	Male	33 (72%)
Laterality, n		
	Right	27 (59%)
	Left	19 (41%)
Etiology, n		
	Parapneumonic	21 (45%)
	Empyema	16 (34%)
	Reactive, intra-abdominal process	2
	Esophageal perforation	2
	Atelectasis	1
	Pleuritis	1
	Malignancy	1
	Unknown	2

Thrombolytic therapy

**Figure 1:**
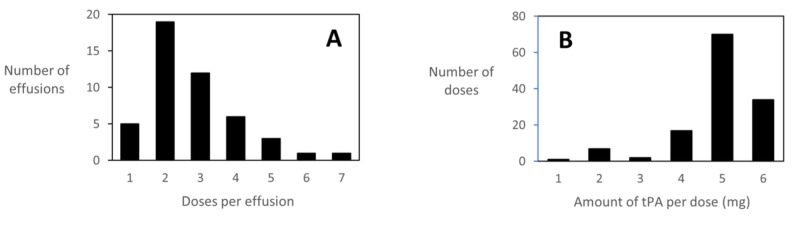
Summary of tPA Dosing for Management of 47 Complex Pleural Effusions A) Number of effusions treated with each number of doses of tPA. Note that five effusions were treated with only one dose of tPA. B) Number of times each amount of tPA was used per dose. tPA: tissue plasminogen activator

Chest tube sizes for initial drainage ranged from 8 to 14 Fr, with the large majority of catheters 8 or 10 Fr (Table 2). A second chest tube was placed in 17 cases, and a third chest tube was placed in one. Fibrinolytic therapy with tPA was delivered 131 times (average 2.7 per patient) in doses ranging from 1 to 6 mg (Figure 1). Sixty-six percent of effusions received two or three doses and 92% of the doses were 4 to 6 mg. For the vast majority of doses, the target dwell time of 12 hours was achieved (Table 3). However, on nine occasions, symptoms such as pressure and dyspnea prompted nursing to unclamp the tubes earlier.

**Table 2:** Chest Tube Size

Chest Tube Size (French)	First Tube (N)	Additional Tube (N)
8	30	11
10	16	5
12	0	1
14	1	0
20	0	1

**Table 3:** Dwell Time

Dwell Time (hours)	Number of Doses
5	1
6	4
7	1
10	1
11	2
12	115
14	1
Not documented	6

Chest tube drainage

**Figure 2:**
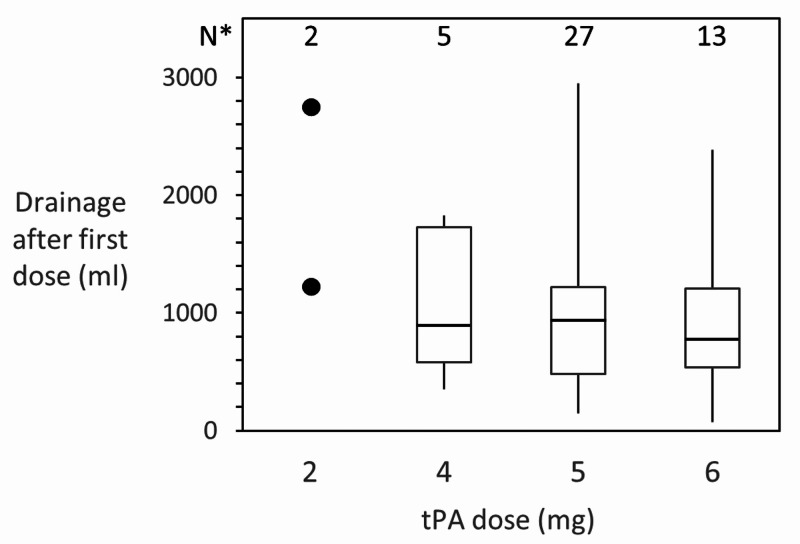
Volume of Pleural Fluid Drained After the First Dose of tPA Range, median and interquartile range for drainage volume measured from when the chest tube was unclamped to when the second dose was given. For patients given only one dose, this is the first 24 hours after the tube was unclamped. Two patients were given 2 mg, with drainage recorded as 460 ml and 3500 ml. * Number of patients in each group. tPA: tissue plasminogen activator

**Figure 3:**
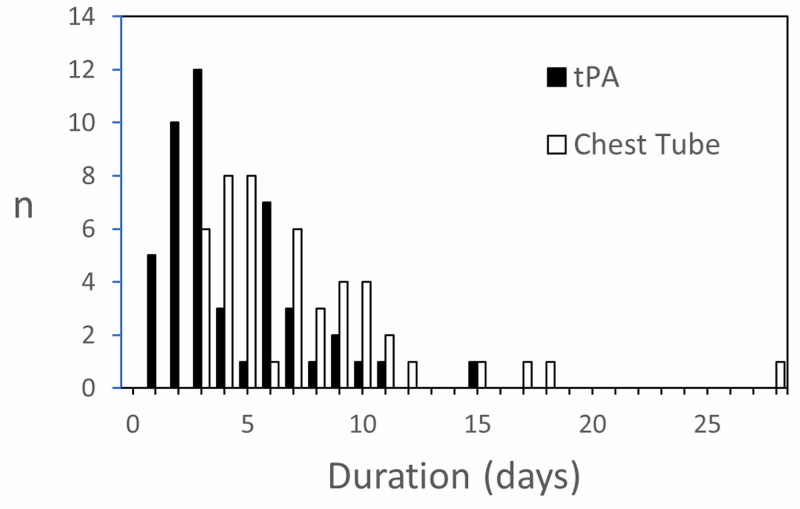
Duration of Therapy Filled bars are for time from the first dose of tPA to the last. Open bars are for time from the first dose of tPA to when the last chest tube was removed. n=number of effusions. tPA: tissue plasminogen activator

Drainage following tPA seemed dose-independent (Figure 2). Duration of therapy was most often three days and 66% of effusions had therapy completed within five days (range 1-15 days, Figure 3). Chest tube drainage was complete by eight days in 68% of cases, and by 11 days in 89% of cases. In one case, chest tube drainage continued for 28 days.

Outcome

**Figure 4:**
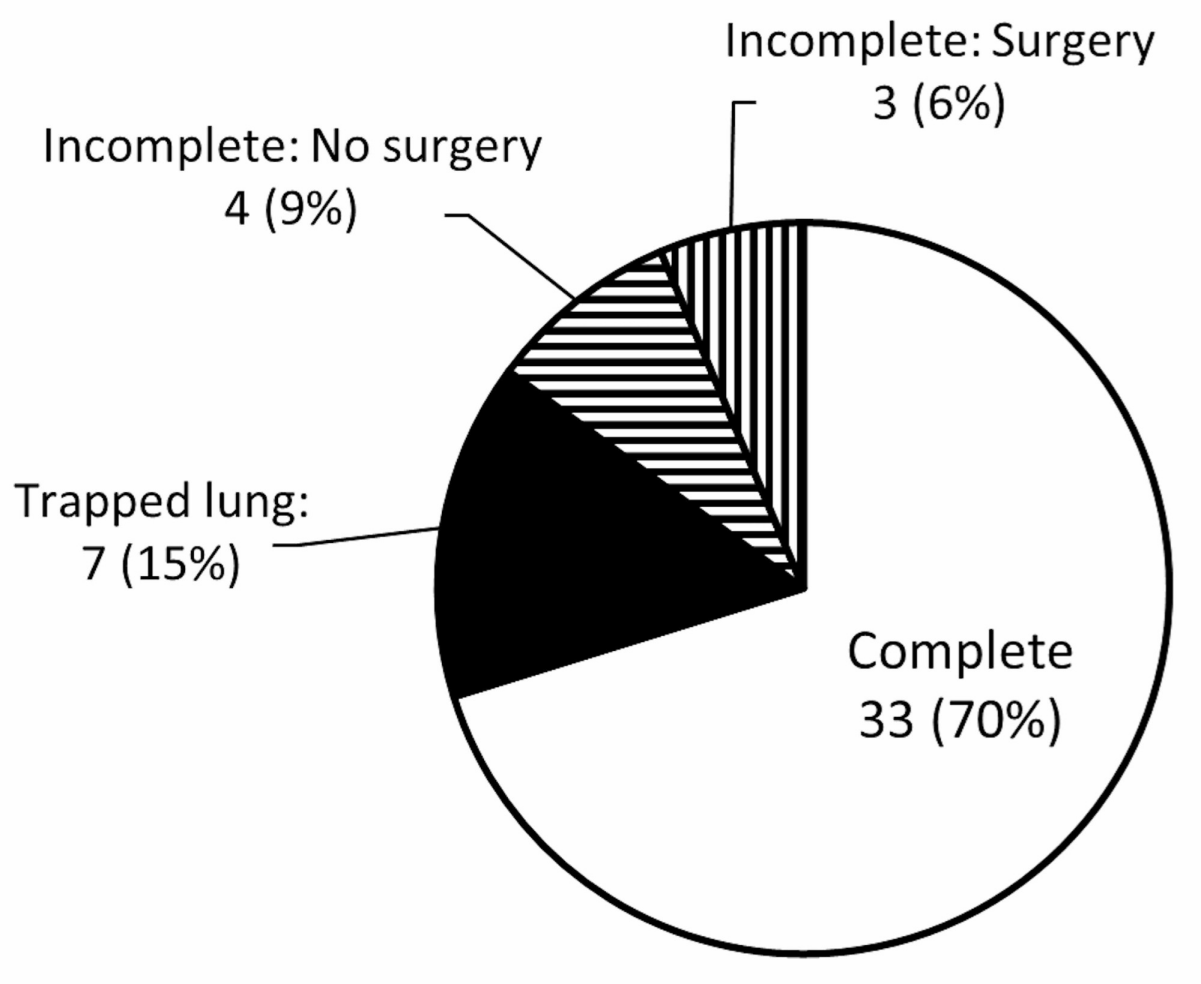
Outcomes of tPA Therapy for 47 Pleural Effusions tPA: tissue plasminogen activator

Post-treatment chest CT scans were available for 41 effusions (87%). For the remaining six, assessment of treatment success was made based on chest x-rays. Most effusions (70%) were assessed to have been completely drained (Figure 4). Of the remaining 30%, half had a trapped lung and the other half had incomplete drainage. Only three cases, half of the effusions with incomplete drainage, underwent decortication. Complete drainage was associated with the shortest symptom duration while trapped lung was associated with the longest (Table 4).

**Table 4:** Symptom Duration by Outcome

Outcome	N	Symptom Duration, days Mean (Range)
Complete drainage	33	6 (1 – 21)
Trapped lung	7	12 (2 – 30)
Incomplete drainage, no surgery	4	5 (2 – 7)
Incomplete drainage, surgery	3	12 (7 – 21)

## Discussion

The goal of intervention for patients presenting with an exudative pleural effusion is to achieve effective drainage with complete lung expansion and relief of symptoms. While decortication can accomplish this goal quickly, an attractive alternative is intrapleural fibrinolytic therapy. If successful, it avoids the morbidity and cost associated with surgery. If unsuccessful, it delays necessary surgery and prolongs hospitalization. We reviewed our experience with a simple regimen of tPA alone with a prolonged dwell time and found that it was successful in the majority of cases, although in some cases treatment duration was prolonged.

Intrapleural fibrinolytic therapy was first described by Tillet and associates in 1951, who reported using streptokinase and streptococcal deoxyribonuclease (DNase) to facilitate pleural drainage in patients with empyema [3]. Streptokinase, and fibrinolytics in general, dissolve fibrinous clots and membranes to prevent fluid sequestration while DNase destroys extracellular DNA, which is thought to help prevent biofilm formation and reduce fluid viscosity. Subsequently, several small controlled studies reported the efficacy of streptokinase and urokinase for improving pleural drainage [4-8]. The limitations of these small studies led to the first Multicenter Intrapleural Sepsis Trial (MIST-1), a large randomized placebo-controlled trial of fibrinolytic therapy published in 2005. MIST-1 found that there was no benefit with streptokinase in radiographic outcomes, need for surgery, survival, or length of hospital stay, but that there were more adverse events [12]. These findings were later supported by a meta-analysis [13]. Although the MIST-1 trial found no benefit, the proportion of enrolled patients who had loculated pleural effusions was low. Therefore, because of otherwise strong clinical and laboratory evidence, interest in intrapleural fibrinolytic therapy continued and research efforts shifted toward the use of recombinant tPA, a direct-acting fibrinolytic agent [14].

There have been several studies supporting the use of intrapleural tPA in adults, however there is wide variation in the number of enrolled patients, number of daily doses, the dose used, and the dwell time of tPA in the pleural space [15-18]. In 2011, the second Multicenter Intrapleural Sepsis Trial (MIST-2) included a comparison of tPA with DNase [9]. MIST-2 found that a twice-daily combination of 10 mg of tPA and 5 mg of DNase with a dwell time of one hour produced a greater decrease in pleural opacity by day seven, decreased surgery referral, and reduced hospital stay relative to double placebo, DNase, or tPA alone [9]. In 2014, Piccolo and colleagues reported improved clearance of pleural opacities in a retrospective series of 107 patients from eight institutions treated with the same combined regimen [10]. In 2016, Mehta and colleagues reported good results in 51 of 55 patients treated with three days of once-daily intrapleural tPA/DNase (10 and 5 mg respectively) and one-hour dwell time [11]. As a result of these studies, current practice for intrapleural therapy for complex pleural effusions is frequent dosing with a combination of tPA and DNase and short dwell times. Because these patients are often distributed across the hospital on different nursing units with varying degrees of expertise with chest tube care, this approach can require significant time commitment from providers, limiting its feasibility. In our experience, based on a serendipitous observation, once-daily therapy with tPA alone and a prolonged dwell time of 12 hours achieved effective drainage in the large majority of cases. This strategy is logistically simpler and less work-intensive, and therefore much more feasible.

For tPA to be a viable alternative to standard surgical therapy, it should achieve similar results with less morbidity and cost. Thoracoscopy is usually associated with minimal morbidity and short hospital stays but is inadequate in the setting of trapped lung, which generally requires thoracotomy for complete decortication. Our results demonstrate that a short course of tPA with a prolonged dwell time achieved the goals of therapy in the large majority (82.5%) of patients who did not have trapped lung, suggesting that it is a viable alternative to thoracoscopy. We also found that tPA was beneficial in those patients with trapped lung because it enabled rapid diagnosis. As expected, symptom duration before presentation was longest in patients with trapped lung, but this factor alone is insufficient for diagnosis. Rapid diagnosis facilitates prompt decision making between proceeding with the more invasive thoracotomy or delaying surgery to proceed electively, when the peel may be more mature and comorbidities are optimized. Incidentally, we found that clearing the pleural space with tPA before decortication seemed to make decortication easier and more straightforward.

Besides overall effectiveness, there are several other important findings in these results. First, intrapleural tPA was safe and well-tolerated. There were no instances of hemodynamically significant pleural bleeding or coagulopathic bleeding from other sites to indicate systemic absorption. Therapy was not painful, although some patients with very large effusions experienced pressure during the dwell time (typically after 8-10 hours), prompting early unclamping of the chest tube. Second, treatment was effective despite the use of small-bore catheters. This is consistent with other reports on the use of pigtail catheters for pleural drainage and is important because these catheters can be inserted with image guidance, an easier and safer alternative to insertion of large-bore chest tubes at the bedside. This reduces the risk of procedure-related morbidities, such as pulmonary parenchymal, liver, or spleen injury. Third, effectiveness seemed independent of tPA dose. As reviewed above, MIST-2 and subsequent trials used a higher dose of tPA (10 mg). The lower dose used in this study, and elimination of DNase, add up to substantial cost savings. In addition, this suggests that future trials can explore the effectiveness of even 2 mg tPA (the unit dose).

**Figure 5:**
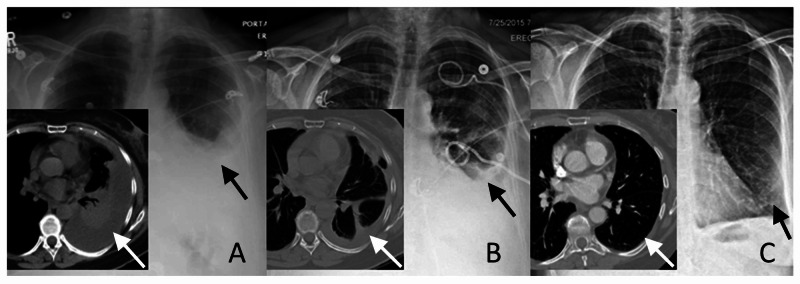
Example of Incomplete Drainage Without Surgery Representative chest x-rays with CT images inset. A 66-year-old woman presented with several weeks of dyspnea and chest pain. A) Chest x-ray suggested a moderate pleural effusion. Chest CT showed left lower lobe atelectasis and consolidation with a parapneumonic effusion. B) A pigtail catheter was placed and she was treated with three doses of tPA. Chest x-ray at completion of therapy showed persistent opacification. Chest CT demonstrated a small residual effusion with a small loculated pocket and with associated pulmonary parenchymal consolidation. The decision was made to conclude therapy and not proceed with surgery. C) Follow-up imaging at one month shows complete resolution of parenchymal and pleural abnormalities. tPA: tissue plasminogen activator.

Objective determination of treatment outcome is essential to making valid conclusions about treatment success. Defining treatment failure as referral to surgery, as has been done in many published trials of fibrinolytic therapy, is suboptimal because this is a clinical decision that is subjective and therefore open to bias and individual clinician preference. (Of the seven patients in our study with incomplete drainage, only three went on to surgery.) Another metric has been clearance of opacification on chest x-ray. We used chest CT for outcome assessment in most of our patients because persistent opacification on chest x-ray may be secondary to atelectasis and/or consolidation, not residual fluid. This is common in patients with parapneumonic effusion and should not be considered treatment failure. Surgical therapy does not treat atelectasis and consolidation, and if the effusion is drained, these findings resolve spontaneously with time (Figure 5). A second benefit of chest CT is that if residual loculated fluid is identified, a decision can be made to either continue tPA through the existing chest tube, insert a new image-guided tube for further therapy, or proceed with decortication. In this study, additional tubes were used in 18/47 cases (38%).

There are several considerations that limit the ability to draw conclusions from this study. Most important is that it is a retrospective analysis of a group of patients managed on an individual basis and without a clearly defined and implemented protocol. Thus, there is considerable uncontrolled variability in many factors that affect outcome. For example, criteria for determining when to repeat tPA and when to remove chest tubes were not defined in advance. in addition, although chest CT was used to evaluate treatment success or failure in most patients, the application was not uniform and objective measurement of residual effusion volume was not employed. Chest CT should be the gold standard for outcome assessment and simple techniques for quantitation of effusion volume based on CT have been published [19]. The retrospective approach also makes it difficult to determine the impact of tPA therapy on hospital length of stay independent of duration of therapy. Some patients were discharged as soon as the chest tube was removed, while in others chest tubes were left in place while patients were kept hospitalized for other reasons. A prospective protocol defining criteria for repeat treatment, chest tube removal, and determination of treatment failure should streamline therapy, shortening duration of therapy and hospital stays. Another important limitation is that long-term follow-up was not available for many patients, so that it is not possible to know if the short-term successes translated into long-term good outcomes.

## Conclusions

Our study suggests that a short course of low-dose tPA with a longer dwell time and without DNase may be equally, if not more, effective than combination therapy of tPA and DNase with frequent dosing and short dwell times. Our strategy is more feasible and less expensive, but treatment can be prolonged. Based on these findings, we are proceeding with a prospective evaluation.

## Appendices

**Table 5:** Microorganism Cultured in Pleural Fluid

Microorganism		n
	Streptococcus viridans	6
	Methicillin-resistant Staphylococcus aureus	2
	Methicillin-sensitive Staphylococcus aureus	1
	Fusobacterium nucleatum	1
	Escherichia coli	1
	Klebsiella pneumoniae	1
	Peptostreptococcus	1
	Eikenella corrodens	1
	Corynebacterium	1
	Enterococcus faecalis	1

**Table 6:** Presenting Symptoms

Symptom		n
	Dyspnea	31
	Cough	13
	Pleuritic Chest Pain	12
	Fever	10
	Chest Pain	7
	Flank Pain	4
	Nausea/Vomiting	3
	Fatigue	2
	Obstructed PleurX® Catheter	2
	Loss of Appetite	2
	Abdominal Pain	2
	Back Pain	1
	Hemoptysis	1
	Dizziness	1
